# SARS-CoV-2 breakthrough infection incidence, risk factors, and antibody correlates in Belgian nursing homes residents and staff (2021–2022)

**DOI:** 10.1186/s13690-026-01939-7

**Published:** 2026-06-24

**Authors:** Liselore De Rop, Eline Meyers, Natalja Van Biesen, Tine De Burghgraeve, Marina Digregorio, Anja Coen, Nele De Clercq, Laëtitia Buret, Samuel Coenen, Els Duysburgh, Beatrice Scholtes, Piet Cools, Stefan Heytens, Jan Y Verbakel

**Affiliations:** 1 Department of Public Health and Primary Care, LUHTAR, Leuven Unit for HTA Research, KU Leuven, Leuven, 3000 Belgium; 2 Department of Diagnostic Sciences, Faculty of Medicine and Health Sciences, Ghent University, Ghent, 9000 Belgium; 3 Department of General Medicine, Faculty of Medicine, Research Unit of Primary Care and Health, University of Liège, Liège, 4000 Belgium; 4 Department of Public Health and Primary Care, Faculty of Medicine and Health Sciences, Ghent University, Corneel Heymanslaan 10, Ghent, 9000 Belgium; 5 Department of Family Medicine & Population Health, Faculty of Medicine and Health Sciences, University of Antwerp, Antwerp, 2000 Belgium; 6 Department of Epidemiology and Public Health, Sciensano, Brussels, 1000 Belgium; 7 Nuffield Department of Primary Care Health Sciences, NIHR Community Healthcare Medtech and IVD Cooperative, University of Oxford, Oxford, OX1 2JD UK

**Keywords:** Nursing home residents, COVID-19 vaccine, Breakthrough infection, Antibody response, Long-term care facilities, Antibody threshold, Correlate of protection

## Abstract

**Background:**

To characterize real-world COVID-19 vaccine-induced protection, we assessed breakthrough infections and protective antibody thresholds among fully vaccinated nursing home residents (NHR) and staff (NHS).

**Methods:**

We conducted a cohort study to assess infection rates and risk factors of symptomatic breakthrough infections in 1605 NHR and 1200 NHS (2021) and 483 NHR (2022) in Belgium using multivariable Cox proportional hazards models adjusted for potential confounders. Additionally, a case-control analysis was used to compare pre-infection and peak vaccine response antibody levels between breakthrough cases and matched controls. Receiver Operating Characteristic (ROC) and net benefit models were fitted to evaluate the protective antibody thresholds in clinical decision making.

**Results:**

In 2021, breakthrough infection incidence was 10.7 (95%CI 8.6–13.2) per 100 person-years in NHS and 2.4 (95%CI 1.59–3.46) in NHR. This rose to 10.6 (95%CI 8.3–13.3) in NHR in 2022. Prior infection was protective in NHS (hazard ratio (HR) 0.25, 95%CI 0.13–0.46) and NHR (HR 0.2, 95%CI 0.05–0.87). In NHS, younger age (HR 1.02, 95%CI 1.01–1.04) and Delta circulation (HR 9.52, 95%CI 2.65–34.21) and in NHR, Omicron BA.5 circulation (HR 17.95, 95%CI 2.35-137.13) increased infection risk. Breakthrough cases had lower pre-infection and peak vaccine antibody levels than controls. Protective antibody thresholds were identified and were higher for infections during Omicron than Delta. Using these antibody thresholds to guide vaccination adds value at low risk thresholds, though net benefits were modest.

**Conclusion:**

Breakthrough infections affected a minority of vaccinated NHR and NHS. Risk depended on age, prior infection, and circulating variants. Lower antibody levels in cases support their protective role, though thresholds are variant-specific. Our findings support tailored vaccine strategies in this vulnerable population.

**Supplementary Information:**

The online version contains supplementary material available at https://doi.org/10.1186/s13690-026-01939-7.

**Table:** 

Text box 1. Contributions to literature
• This study provides real-world evidence on COVID-19 infections after vaccination among nursing home residents and staff in Belgium.
• It identifies antibody levels associated with protection, contributing to the search for reliable markers of immunity.
• Findings show that infection risk and protective thresholds varied across virus variants, underlining the importance of ongoing evaluation.
• By linking antibody thresholds to decision models, the study informs vaccination and booster strategies for vulnerable groups.

## Background

Long-term care facilities, such as nursing homes (NH), were disproportionately affected by the COVID-19 pandemic. These facilities house a vulnerable population, characterized by advanced age and multiple underlying health conditions, making them particularly vulnerable to severe disease and mortality caused by SARS-CoV-2. In Belgium, nearly 57% (*n* = 12,447) of all COVID-19 deaths during the first year of the pandemic occurred among NH residents (NHR) [1], highlighting the need to prioritize this population for vaccination. As a response, vaccination was initiated in Belgian NH on 28 December 2020 [2]. However, due to the underrepresentation of NHR in clinical trials and limited real-world data, uncertainties endured regarding the level of protection vaccination would offer against hospitalization and mortality in this high-risk population [3]. To address this, the SCOPE study, a national longitudinal prospective seroprevalence study, was initiated in 69 NH across Belgium [4]. This study collected serological and clinical data from NHR and NH staff (NHS) following primary COVID-19 vaccination and, where applicable, during subsequent booster vaccination periods.

Although national COVID-19 vaccination coverage in the general Belgian population exceeded 80% by 11 August 2021, SARS-CoV-2 infections persisted in 2021, including a moderate third wave in mid-February and a large fourth wave in early October [5]. Various factors, including the emergence of highly transmissible variants, waning vaccine-induced immunity, and changes in preventive measures, may have contributed to these breakthrough infections [5]. To this end, identifying predictors of breakthrough infections is essential to optimize protective strategies, particularly in vulnerable populations such as NHR. Furthermore, understanding how antibody levels correlate with protection against COVID-19 is crucial for accurately assessing the degree of immunity, as identifying thresholds for future booster strategies and as early indicators of vulnerability is key.

Other studies have investigated SARS-CoV-2 breakthrough infections and proposed antibody thresholds associated with protection, yet, the majority of these studies were conducted in younger adults or healthcare workers [6–14]. Therefore, breakthrough infections in NHR including assessment of antibody levels prior to infection for identification of protective thresholds, remains poorly investigated. Nevertheless, this knowledge is crucial in refining vaccination strategies, guiding public health policies, and improving risk assessment in this high-risk population. By analyzing breakthrough infections as well as antibody concentrations both prior to infection and at the peak of antibody vaccine response (two months post-vaccination), this study contributes to evidence-based decision-making for future pandemics and the long-term protection of vulnerable populations in NH.

Therefore, the objectives of this study were to:

1. Assess the incidence and predictors of breakthrough infections in fully vaccinated NHR and NHS during the first-year post-vaccination and in NHR during the second year.
2. Compare pre-infection and peak vaccine response SARS-CoV-2 antibody concentrations between individuals with and without breakthrough infection, and explore antibody thresholds for protection.

## Methods

### Study design and population

**Fig. 1:**
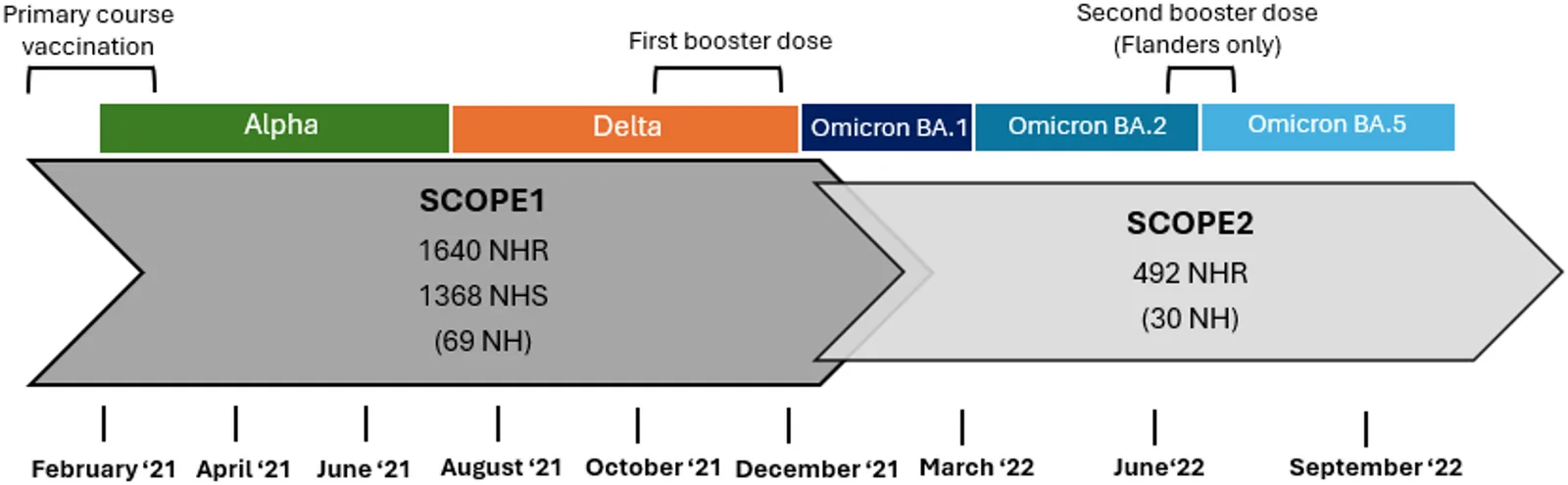
Timeline with an overview of the study visits (February 2021 - September 2022), dominant SARS-CoV-2 variants and COVID-19 vaccination rounds in Belgian nursing homes (NH) [18]. The total number of participants included in SCOPE1 and SCOPE2 is presented. NHR: nursing home residents; NHS: nursing home staff

This study combines a prospective cohort design (clinical characterization) with a matched case–control design (serological characterization). The data used in this study was collected within the SCOPE study, a longitudinal prospective national seroprevalence study in Belgian NHR and NHS, which has been previously described [15, 16]. The first part of the study, referred to as SCOPE1, ran between February and December 2021 with visits every two months collecting Dried Blood Spot (DBS) and clinical data in NHR and NHS. The study was extended from December 2021 to September 2022 in (a smaller subset of) NHR only, referred to as SCOPE2, with collection of DBS and clinical data every three months. An overview of the study timeline is presented in Fig. 1. The sample size calculation has been described previously [16]. A random sample of 69 NH was recruited across Belgium in proportion to the population per region (Flanders/Brussels/Wallonia) and number of NH beds per province. A total of 24 NHR and 20 NHS were randomly selected within every NH. A more detailed recruitment strategy was previously described [16]. In SCOPE2, a stratified random sample of 30 NH that had already participated in SCOPE1 was selected, as previously described [17]. Participants for SCOPE2 were selected randomly and a breakthrough infection in SCOPE1 was not an exclusion criteria for participation in SCOPE2.

In this study’s analysis, SARS-CoV-2 breakthrough infections were assessed in fully vaccinated NHR/NHS. Participants were considered fully vaccinated 14 days after completing primary course vaccination (BNT162b2 or ChAdOx1 or mRNA-1273 in two-dose regimen or one dose of Ad26.COV2.S). A SARS-CoV-2 breakthrough infection was defined as a self-reported positive COVID-19 test result (PCR, antigen test, and/or CT-scan) during follow-up, occurring at least 14 days after primary COVID-19 vaccination, with at least one COVID-19-specific-like symptom (see section *Questionnaires*) reported within seven days before or after the positive test result. Breakthrough infections were excluded if another positive test occurred within a 90-days timeframe prior to infection, to prevent inclusion of cases with persistent viral shedding [19].

### Ethical considerations

This study was approved by the Ethics Committee of the Ghent University Hospital (reference number BC-08719) and conducted according to the principles outlined in the Declaration of Helsinki. Each participant or their legal representative (e.g., for NHR with dementia) signed an informed consent form after being informed about the goal of the study and the study procedures.

### Data collection

#### Dried blood spot collection

At every test round, trained study staff collected capillary blood with a fingerprick using an 18G lancet 1.8 mm depth (SARSTEDT Ag & Co., Nümbrecht, Germany) and collected it on a DBS saver card (EUROIMMUN, Lübeck, Germany). Minimally four 6 mm-diameter circles were saturated with blood until saturation was visible on the back of the card and left to air dry for at least one hour. Samples were stored at − 20 °C until analysis [20].

#### S1-RBD IgG antibody concentration

The SARS-CoV-2 anti-spike 1 receptor binding domain (S1-RBD) IgG ELISA assay (ImmunoDiagnostics, Hong Kong), an assay calibrated with the World Health Organization (WHO) International standard (NIBSC 20/136) [21], was used to quantify the antibody concentrations. The DBS samples were prepared and processed as previously described [22]. Briefly, a spot of 6 mm diameter was punched from the DBS and placed into 250 µL of 1x S1-RBD IgG ELISA buffer. After a one-hour incubation at 37 °C, the extract was diluted 100-fold and transferred to a S1-RBD-coated well plate. The remainder of the procedures followed the manufacturer’s ELISA assay instructions, with an optical density (OD) reading at 450 nm using the Behring ELISA Processor III (Siemens AG, Munich, Germany). Samples with OD higher than that of the highest standard value were retested using a 10- and/or 1000-fold dilution based on the expected range. Antibody concentrations (IU/mL) were calculated using a four-parameter logistic (4PL) regression curve (GraphPad Prism version 10.3.0 (GraphPad Software Inc., San Diego, CA, USA) based on a set of SARS-CoV-2 antibody standards provided in the kit. A seropositivity cutoff value of 26 IU/mL was experimentally determined [22].

#### Questionnaires

At baseline and during each testing round, NHS were required to complete an online questionnaire (LimeSurvey version 3.22). For NHR, the head nurse(s) of the NH completed the questionnaires on their behalf, based on the medical file of the NHR. At baseline, data were collected on sociodemographic characteristics, including age, sex, and job type (for NHS). Additional information was gathered on COVID-19-relevant comorbidities [23] such as cardiovascular disease, diabetes, hypertension, immunosuppression, severe renal, lung, or cardiac disease, and active cancer. Additionally, during each testing round, participants were asked to report their COVID-19 vaccination status (number of doses, dates, and vaccine type) and any confirmed SARS-CoV-2 infections (determined by PCR, antigen testing, or CT scan, including the date of diagnosis). Participants were asked whether they experienced any of the following symptoms: cough, dyspnoea, chest pain, loss of taste, loss of smell, worsening of chronic respiratory conditions (e.g., COPD, asthma, chronic cough, etc.), fever, myalgia, fatigue, runny nose, throat ache, headache, loss of appetite, diarrhoea, confusion, and collapse. In the event of an NHR’s death during the follow-up period, information on the date of death and its cause (COVID-19-related or non-COVID-19-related) was recorded.

#### SARS-CoV-2 variants

In this study, molecular data on the specific SARS-CoV-2 variant causing the breakthrough infections was not collected. To evaluate the impact of different variants of concern (VOC) on breakthrough infections, we utilized data from Sciensano, the national public health institute of Belgium, which monitors the prevalence of circulating variants over time [18]. In this way, we used data on the dominant VOC at the time of breakthrough infection. The following periods were assigned to each VOC: Alpha (15/02/2021–27/06/2021), Delta (28/06/2021–30/12/2021), Omicron BA.1 (31/12/2021–03/03/2022), Omicron BA.2 (04/03/2022–14/06/2022), and Omicron BA.5 (15/06/2022–25/10/2022). The term Omicron includes all three sublineages (Omicron BA.1, Omicron BA.2, Omicron BA.5).

### Statistical analysis

Participant characteristics were analyzed descriptively for participants with and without a breakthrough infection. Median age was calculated with an interquartile range (IQR), and minimum and maximum. The categorical variables were described using absolute (n) and relative frequencies (%). Missing data is reported per variable.

#### Predictors of breakthrough infection

The breakthrough infection proportion with 95% confidence intervals (CI) was calculated as the number of breakthrough infections over the total number of included participants for NHS and NHR in SCOPE1 and NHR in SCOPE2. Additionally, we determined the incidence rate per 100 person-years with 95% CI. Follow-up time started 14 days after completion of the primary course vaccination until the breakthrough infection or last available follow-up. Hazard ratios (HR) with 95% CI were calculated for breakthrough infection using Cox proportional hazard models, adjusted for the following a priori selected predictors: age, sex, comorbidity (presence of comorbidity in NHS and type in NHR), self-reported history of SARS-CoV-2 infection prior to primary course vaccination, and the VOC. The VOC was modeled as a time-dependent variable. This approach allowed for the estimation of HR specific to each VOC relative to other periods. To account for the potential correlation between participants within the same NH, we employed a marginal model with a cluster-robust sandwich estimator for the standard errors. Event counts and person-time are presented alongside the Cox model results to facilitate interpretation; hazard ratios were estimated directly from the Cox proportional hazards models. We assessed HR in NHR and NHS during SCOPE1, and in NHR during SCOPE2. Analyses for NHR in SCOPE1 and SCOPE2 were conducted separately due to differences in study design (e.g., follow-up frequency, and time frame) and the smaller sample size in SCOPE2. Due to collinearity and model instability not all a priori selected predictors could be included in every model. In the NHS model (SCOPE1), the included variables were age, sex, absence (vs. presence) of comorbidities, history of SARS-CoV-2 infection, and the VOC. In the NHR model of SCOPE1, the included variables were age, sex, comorbidity type, and history of SARS-CoV-2 infection. The VOC was not included in this model because of convergence issues during model fitting due to data separation, 25 out of 28 breakthrough infections were associated with a single VOC (Delta). In the NHR model of SCOPE2, the included variables were age, sex, comorbidity type, history of SARS-CoV-2 infection and VOC. The VOC could be included in this model, as its distribution was more balanced and did not cause model instability. However, the immune-related comorbidity could not be included due to data separation, leading to convergence problems. Only seven NHR reported an immune-related comorbidity of which none had a breakthrough infection. For participants who did not experience the event of interest, the censoring date was the date of last available follow-up questionnaire. Complete case analysis was performed. A sensitivity analysis was performed to assess the potential impact of reporting bias of COVID-19-related symptoms on symptomatic breakthrough infections. To do so, we examined predictors of all breakthrough infections, including both symptomatic and asymptomatic cases. An alpha of 0.05 was chosen with a *p*-value less than 0.05 indicating statistical significance. The analyses were performed in R version 4.2.0.

#### Assessment of pre-infection and peak vaccine response antibody concentrations

Pre-infection S1-RBD IgG concentrations were defined as the antibody level measured ≤ 30 days before breakthrough infection. Peak vaccine response S1-RBD IgG concentrations were defined as the antibody concentrations measured during the first visit after the primary vaccination course (April 2021). This was based on previously published observations, as we showed that, of all timepoints in our study (before booster), the timepoint in April 2021 showed the highest concentrations post-primary course vaccination, obtaining a peak response [24]. To assess pre-infection S1-RBD IgG concentrations, subjects with a sample available collected ≤ 30 days before breakthrough infection were selected among all breakthrough cases. This resulted in 48 available samples. Individually matched controls for these samples were searched in a 1:10 ratio, based on participant type (NHR/NHS) vaccination status (number of received doses) and study visit (sampling month)) from a database with previously analyzed samples from the SCOPE study including 3130 samples, collected at different visits. More info on the database used for selection of matched controls can be found in Supplementary Table 1. To assess peak vaccine response S1-RBD IgG concentrations among breakthrough cases, 37 of the 48 previously defined cases had a peak vaccine response S1-RBD IgG sample available. For these 37 cases, another set of controls was searched in a 1:10 ratio, based on the same criteria as before. To assess differences in S1-RBD IgG concentrations between cases and controls, unpaired t-tests were used after log transformation of the S1-RBD IgG concentrations. Geometric means with 95% CI were reported. To assess differences in S1-RBD IgG concentrations between VOC within cases and controls, 2-way ANOVA with Tukey’s multiple comparisons test was applied on the log-transformed data, for which normality was assumed. Receiver Operating Characteristic (ROC) curves were plotted for all pre-infection and peak vaccine response S1-RBD IgG concentrations and separate for breakthrough infection cases/controls during the Delta and Omicron wave. ROC curves were reported with the area under the curve (AUC) and 95% CIs. The optimal thresholds for classification were based on the Youden’s Index and were reported with their corresponding sensitivity and specificity with 95% CI. For this application, sensitivity refers to the ability to detect breakthrough infection in individuals with an S1-RBD IgG level below the optimal threshold and specificity of the assay refers to the ability to identify individuals with an S1-RBD IgG level above the optimal threshold without breakthrough infection [25]. Additionally, net benefit models (separate per variant and pre-infection/peak vaccine response) were fitted to evaluate the clinical utility of the antibody threshold defined by the ROC analysis. To correct for the case-control design which does not reflect the incidence of breakthrough infections in our population, a simulated population of 10,000 individuals was created by resampling log-transformed antibody levels from the original dataset. Infection probabilities were predicted using a logistic regression model and recalibrated to reflect a target infection incidence of 5.7%, based on the breakthrough infection rate in the total population (187/3288). This simulated data was used for decision curve analysis, including the calculation of the net benefit, predicted infection probability and false positive (FP)/true positive (TP) ratio for the antibody thresholds defined by ROC analysis. The net benefit was calculated for the high-risk threshold (the probability of breakthrough infection at which action is desired) that was set to the respective predicted infection probability, and visually represented for all risk thresholds (up to 50%). In decision curve analysis, the net benefit corresponds to the weighed benefit of correctly identifying at-risk individuals (TP) against the harm of incorrectly identifying people (FP) using antibody thresholds as a clinical decision tool for vaccine intervention. The predicted infection probability represents the infection risk for subjects with an antibody level below the antibody threshold, and the FP/TP ratio represents the proportion of individuals incorrectly classified as at-risk for every correctly identified individual at-risk. An alpha of 0.05 was chosen with a *p*-value less than 0.05 indicating statistical significance. All analyses were performed in GraphPad Prism Version 10.3 (GraphPad Software, Boston, Massachusetts USA), except for the net benefit analysis, which was performed in R version 4.5.0.

## Results

### Participants characteristics

**Fig. 2:**
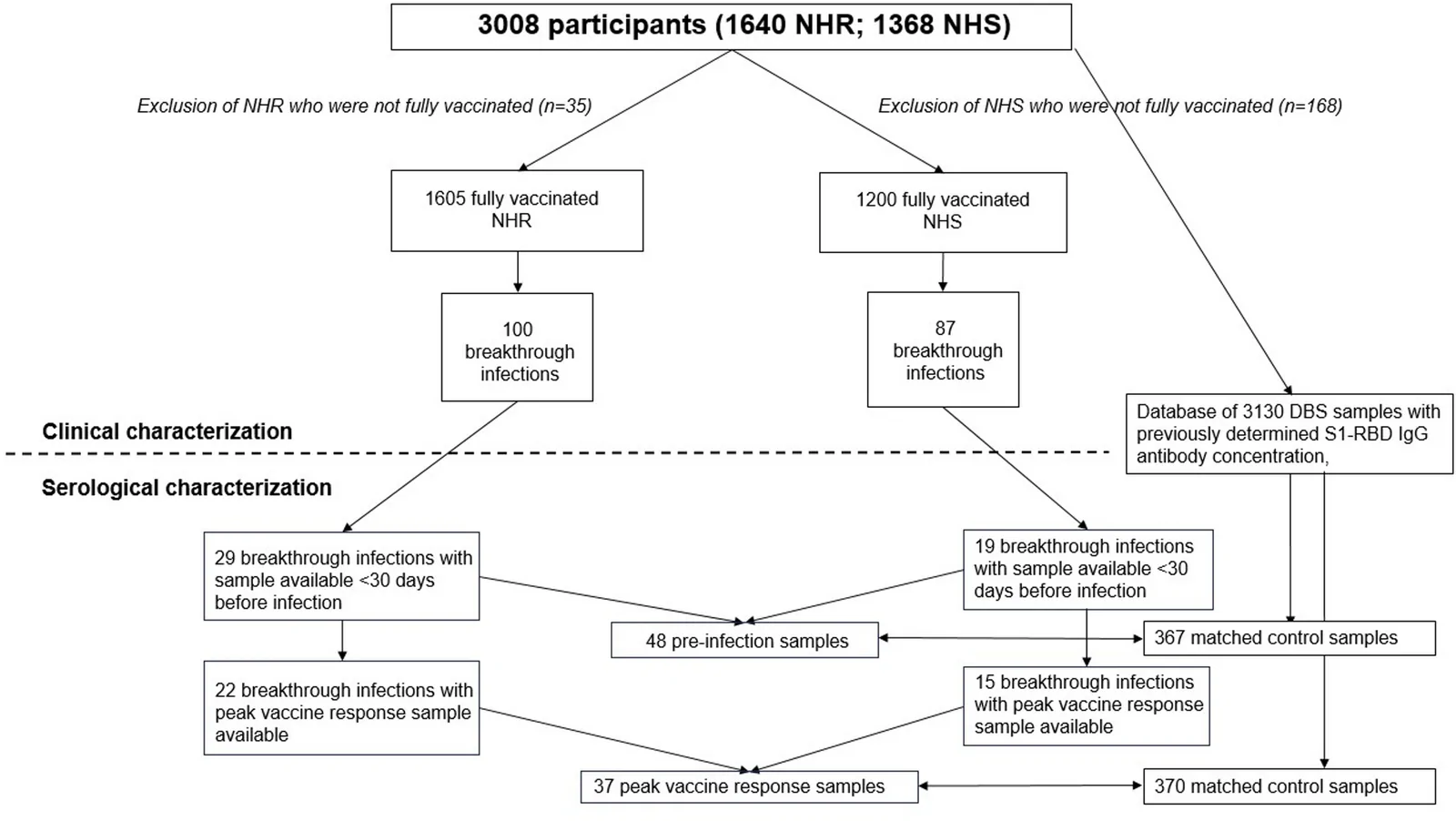
Schematic overview of the participants and samples included in the analysis. Above the dashed line describes the data included in the clinical characterization, below the dashed line presents the data included in the serological characterization. NHR: nursing home residents; NHS: nursing home staff

The participant flow is displayed in Fig. 2. A total of 3008 participants (1640 NHR and 1368 NHS) from 69 NH across Belgium were included in SCOPE1 between February and December 2021. Between December 2021 and September 2022, the study was extended in 492 NHR from 30 NH, as part of SCOPE2. For the analyses in the current publication, only fully vaccinated participants were included, being 1605 NHR and 1200 NHS in the first part and 483 NHR in the second part. For the serological characterization of breakthrough infections, cases with a pre-infection and peak vaccine response sample available were selected. For pre-infection samples, 376 controls were found for 48 cases, with a minimum of one control per case. For peak vaccine response samples, 370 controls were found for 37 cases. The baseline characteristics of all participants included in the current analysis (*n* = 2805; 1605 NHR and 1200 NHS) are shown in Table 1, separated for participants with and without a breakthrough infection. The median age was 88 years (IQR 82–91) in NHR and 44 years (IQR 34–53) in NHS. In NHR, 73% of participants were female, and in NHS, this was 84%. The most prevalent comorbidities in NHR were cardiovascular disease, hypertension and diabetes. In NHS, 20% reported to have a comorbidity, most prevalent hypertension. The majority of NHR and NHS were vaccinated with BNT162b2 (99% in NHR and 98% in NHS).

**Table 1:** Participant characteristics in nursing home residents and staff with and without a breakthrough infection (Belgium, 2021–2022)

Participant characteristics	Nursing Home Residents (*n* = 1605)		Nursing Home Staff (*n* = 1200)	
	**Breakthrough Infection** (*n* = 100)	**No breakthrough infection** (*n* = 1505)	**Breakthrough Infection** (*n* = 87)	**No breakthrough Infection** (*n* = 1113**)**
Age (years)				
Median (interquartile range)	87 (82–90)	88 (82–91)	41 (34–47)	44 (34–53)
Minimum-maximum	48–96	46–104	24–63	18–71
Missing values	2	64	3	68
Sex – *n* (%)				
Female	73 (73)	1092 (73)	74 (85)	932 (84)
Male	26 (26)	359 (24)	12 (14)	159 (14)
Missing values	1 (1)	54 (4)	1 (1)	22 (2)
Type of comorbidity – *n* (%)				
Cardiovascular disease	43 (43)	593 (39)	4 (5)	33 (3)
Diabetes	15 (15)	256 (17)	2 (2)	36 (3)
Hypertension	38 (38)	487 (32)	7 (8)	128 (12)
Severe renal/lung/cardiac disease	13 (13)	160 (11)	0	27 (2)
Immunosuppression	2 (2)	35 (2)	0	22 (2)
Active cancer	10 (10)	59 (4)	0	8 (1)
No comorbidities	24 (24)	466 (31)	73 (84)	884 (79)
Missing values	1 (1)	54 (4)	1 (1)	22 (2)
History of COVID-19 infection– *n* (%)	10 (10)	581 (39)	9 (10)	377 (34)
Missing values	0	0	0	0
Brand of primary vaccine – *n* (%)				
BNT162b2	98 (98)	1494 (99)	85 (98)	1096 (98)
Other (ChAdOx1, mRNA-1273, Ad26.COV2.S)	2 (2)	11 (1)	2 (2)	17 (2)

### Occurrence and incidence of breakthrough infections in nursing home residents and staff

**Fig. 3:**
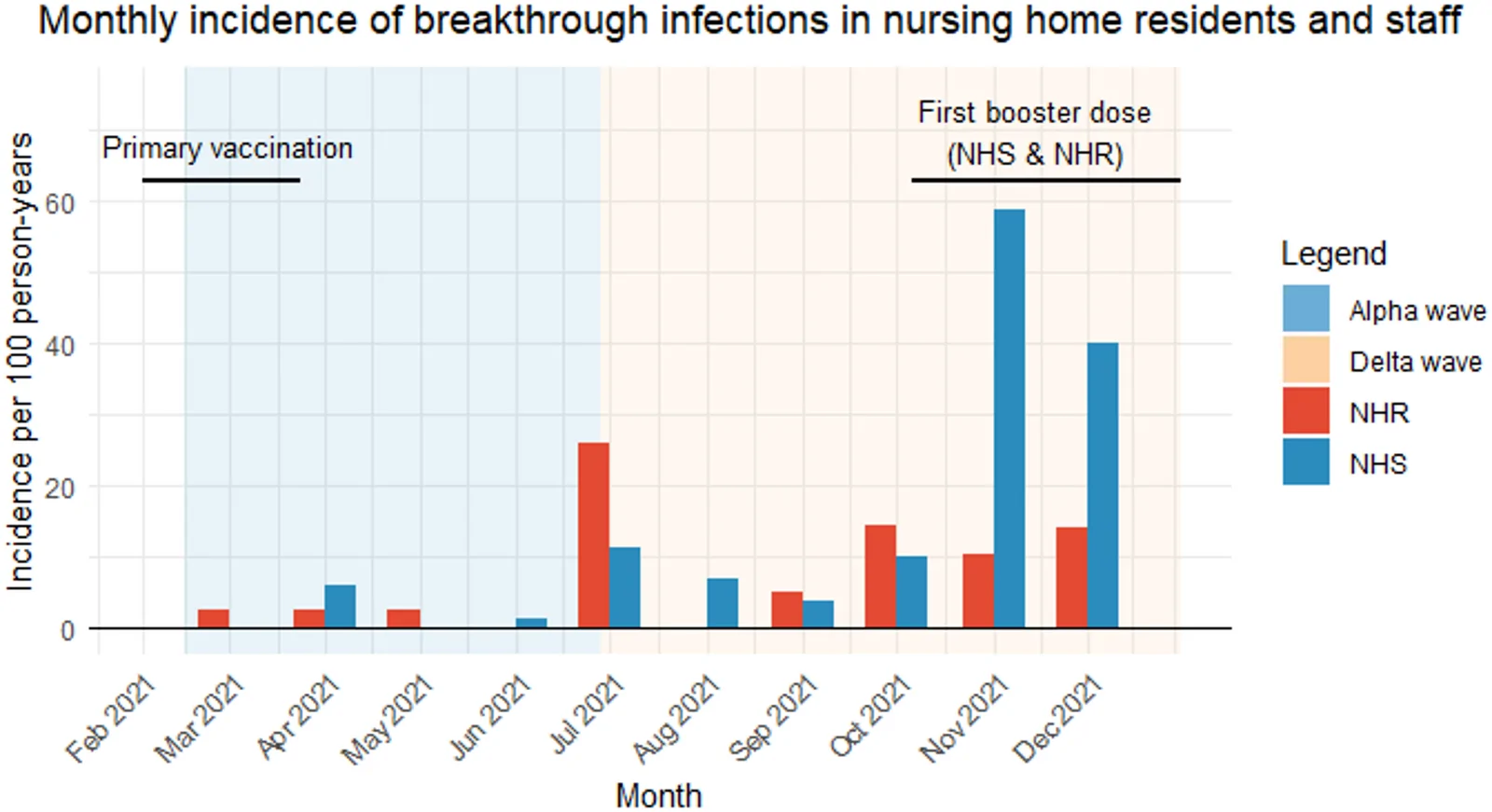
Monthly incidence of breakthrough infections (per 100 person years) in fully vaccinated nursing home residents (NHR, red) and staff (NHS, blue) between February and December 2021, Belgium. A breakthrough infection was defined as a symptomatic self-reported positive test (PCR, antigen test, or CT-scan) occurring ≥ 14 days after completion of the primary COVID-19 vaccination course, with symptoms reported within seven days before or after the positive test. Background shading indicates dominant SARS-CoV-2 variant waves (Alpha and Delta). Horizontal lines indicate timing of the primary vaccination period and first booster campaign

**Fig. 4:**
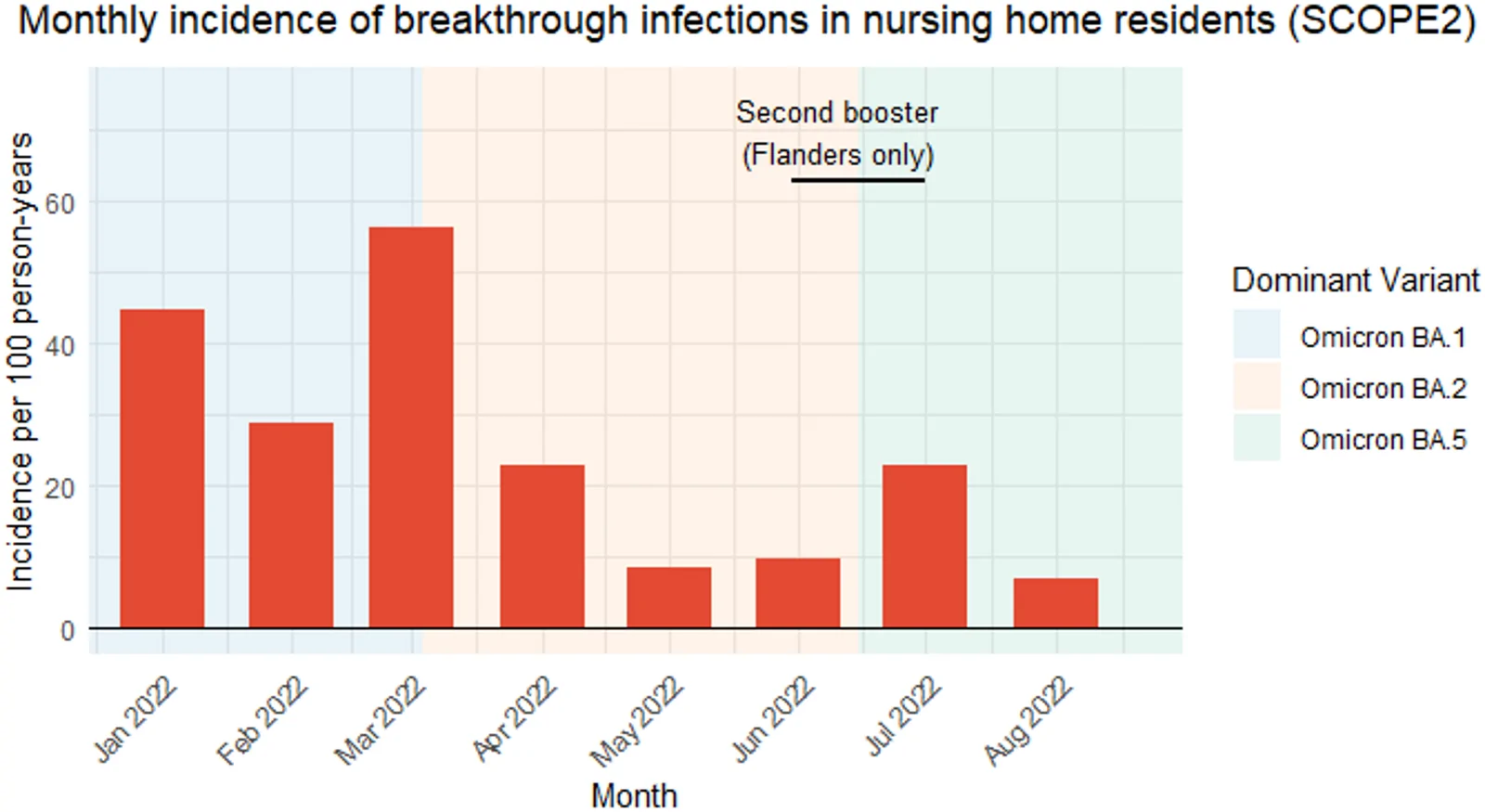
Monthly incidence of breakthrough infections (per 100 person years) in fully vaccinated nursing home residents (NHR) between January and August 2022, Belgium. A breakthrough infection was defined as a symptomatic self-reported positive test (PCR, antigen test, or CT-scan) occurring ≥ 14 days after completion of the primary COVID-19 vaccination course, with symptoms reported within seven days before or after the positive test. Background shading indicates dominant SARS-CoV-2 variant waves (Omicron BA.1, Omicron BA.2, and Omicron BA.5). Horizontal lines indicate timing of the second booster campaign

In SCOPE1, between February and December 2021, 115 (4%, 95%CI 3–5) participants developed a breakthrough infection, including 28 (2%, 95%CI 1–3) fully vaccinated NHR and 87 (7%, 95%CI 6–9) fully vaccinated NHS. The incidence of breakthrough infections was 2.4 per 100 person years (95% CI 1.59–3.46) and 10.7 per 100 person years (95% CI 8.6–13.2) in NHR and NHS, respectively (Fig. 3). Most infections in NHS occurred in November 2021, during which cases were identified in 29 different NH, with a maximum of four infections in the same NH. The median time to infection was 257 days (IQR 174–276) in NHS and 175 days (IQR 146–247) in NHR. During SCOPE2, 72 (15%, 95%CI 12–18) breakthrough infections developed in NHR with an incidence of 10.6 per 100 person years (95% CI 8.3–13.3) (Fig. 4). Most breakthrough infections (*n* = 21) occurred in March 2022, affecting eight different NH, with one NH reporting 10 cases. In this group median time from primary vaccination to infection was 378 days (IQR 346–413). None of the participants reported a second breakthrough infection.

### Symptoms and predictors of breakthrough infections in nursing home residents and staff

The most frequently reported symptom during a breakthrough infection was fatigue (82%) in NHS, whereas in NHR, it was cough (56%). The frequency of symptoms is displayed in Supplementary Table 2. Among NHR who experienced a breakthrough infection (*n* = 102) 17 NHR died from any cause. The median time from breakthrough infection to death was 98 days (IQR 23–126). Five NHR died within 30 days of the breakthrough infection. Among NHR who did not experience a breakthrough infection (*n* = 1503), 309 NHR died from any cause. None of the NHS died.

**Fig. 5:**
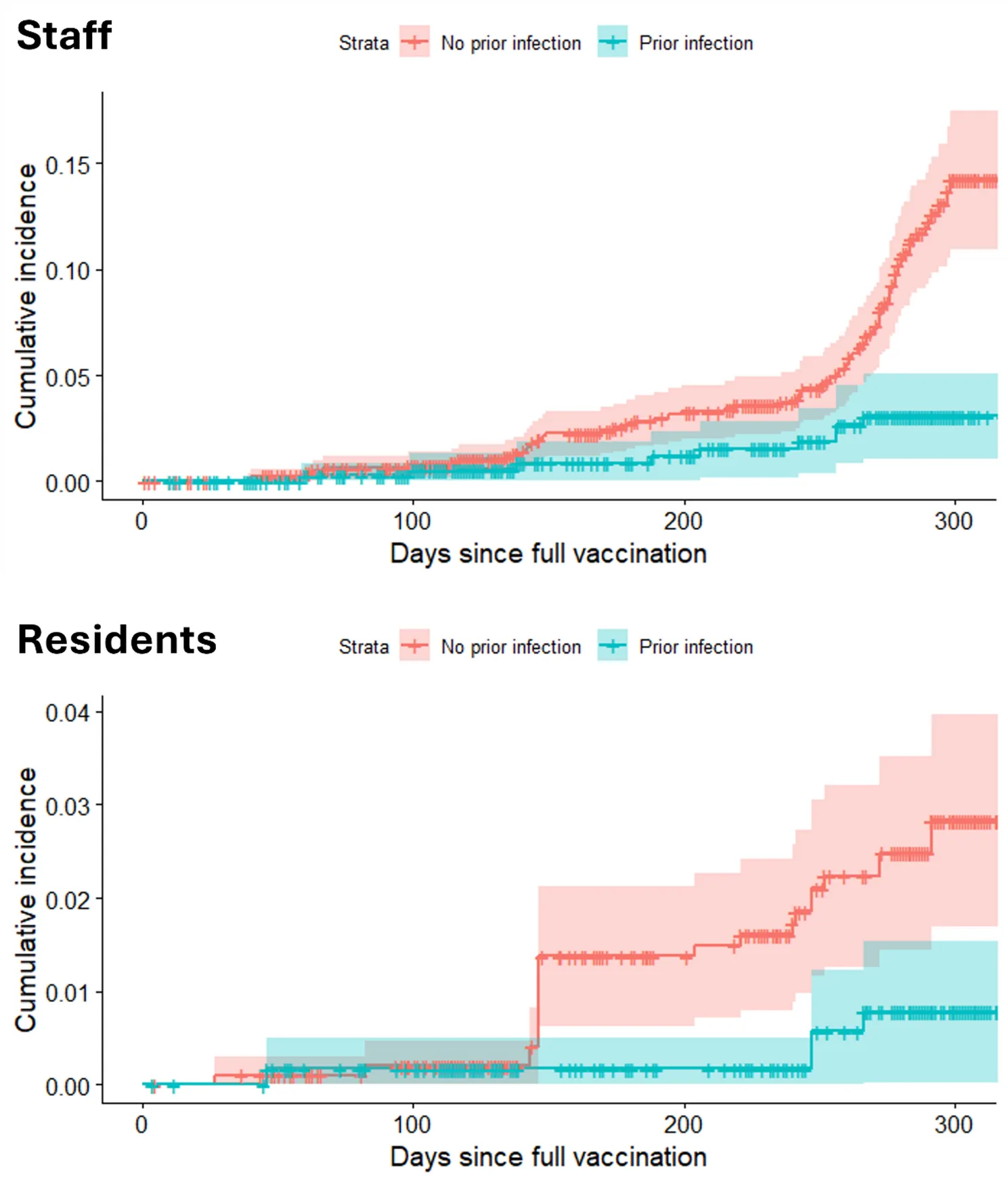
Cumulative incidence of breakthrough infections among fully vaccinated nursing home staff and residents stratified for participants with and without a SARS-CoV-2 infection prior to full vaccination, Belgium (2021–2022). Shaded areas indicate 95% confidence intervals. Full vaccination was defined as 14 days post-primary course

Based on the Cox proportional hazard model of NHS, older age (HR 0.98, 95% CI 0.96–0.99) and a history of SARS-CoV-2 infection prior to primary vaccination (HR 0.25, 95% CI 0.13–0.46) were protective of a breakthrough infection (Table 2; Fig. 5). In NHS, the Delta wave (HR 9.52, 95% CI 2.65–34.21) was associated with a higher risk of breakthrough infection compared to the Alpha wave. In NHR during SCOPE1, we were unable to assess the impact of the different variant waves, because 25 out of 28 breakthrough infections were associated with a single VOC (Delta). Prior infection with SARS-CoV-2 was protective against breakthrough infection in this group (HR 0.2, 95% CI 0.05–0.87; Table 2; Fig. 5). This was also seen in NHR in SCOPE2 (HR 0.10, 95% CI 0.04–0.27; Table 3, Supplementary Fig. 1). In this group, the Omicron BA.5 wave (HR 17.95, 95% CI 2.35-137.13) was associated with a higher risk of breakthrough infection compared to the Omicron BA.1 wave. Additionally, NHR with cancer as a comorbidity were at higher risk of breakthrough infection (HR 3.15, 95% CI 1.44–6.91).

**Table 2:** Predictors associated with a breakthrough infection in nursing home staff^a^ and residents^b^: February – December 2021, Belgium

Predictors	Hazard Ratio	95% Confidence Interval	Events/person years
Nursing home staff^a^ - February to December 2021			
Age, in years	*0.98*	0.96–0.99	-
Male	0.84	0.46–1.51	11 / 117.1
Female	Ref	-	73 / 649.3
No comorbidities	1.05	0.55–2.01	71 / 623.5
≥ 1 comorbidity	Ref	-	13 / 142.9
History of SARS-CoV-2 infection	*0.25*	0.13–0.46	9 / 249.3
No history of SARS-CoV-2 infection	Ref	-	75 / 517.1
Delta wave	*9.52*	2.65–34.21	78 / 425.4
Alpha wave	Ref	-	6 / 341.0
Nursing home residents^b^ - February to December 2021			
Age, in years	0.96	0.91–1.03	-
Male	0.87	0.42–1.80	7 / 278.0
Female	Ref	-	19 / 866.8
Hypertension	0.76	0.40–1.43	7 / 391.5
No hypertension	Ref	-	19 / 753.3
Respiratory disease	1.21	0.53–2.75	4 / 125.0
No respiratory disease	Ref	-	22 / 1019.8
Immune disorder	2.21	0.32–15.51	2 / 27.1
No immune disorder	Ref	-	24 / 1117.7
Diabetes	0.63	0.21–1.91	3 / 202.5
No diabetes	Ref	-	23 / 942.4
Cancer	2.62	0.34–19.94	3 / 49.2
No cancer	Ref	-	23 / 1095.6
Cardiovascular disease	1.04	0.63–1.71	10 / 467.9
No cardiovascular disease	Ref	-	16 / 676.9
History of SARS-CoV-2 Infection	*0.20*	0.05–0.87	3 / 443.6
No history of SARS-CoV-2 infection	Ref	-	23 / 701.2

Hazard ratios with 95% confidence intervals were estimated using multivariable Cox proportional hazards models for breakthrough infection, adjusted for covariates and accounting for clustering at the nursing home level. Event counts and person-time are reported for descriptive purposes only; hazard ratios are not directly calculated from these incidence rates. Hazard ratios shown in italics are statistically significant (*p* < 0.05)

^a^Reference for nursing home staff: female, presence of a comorbidity, no history of SARS-CoV-2 infection prior to primary course vaccination, and a breakthrough infection during the Alpha wave (15/02/2021–27/06/2021). Delta was the VOC during 28/06/2021–30/12/2021. ^b^Reference for nursing home residents: female, no hypertension, no respiratory disease, no immune disorder, no diabetes, no cancer, no cardiovascular disease, and no history of SARS-CoV-2 infection prior to primary course vaccination

**Table 3:** Predictors associated with a breakthrough infection in nursing home residents^a^: January – September 2022, Belgium

Predictors	Hazard Ratio	95% Confidence Interval	Events/person years
Age, in years	1	0.98–1.02	-
Male	0.83	0.50–1.37	19 / 65.2
Female	Ref	-	53 / 196.2
Hypertension	1.22	0.69–2.15	30 / 90.5
No hypertension	Ref	-	42 / 170.9
Respiratory disease	1.24	0.63–2.46	9 / 26.0
No respiratory Disease	Ref	-	63 / 235.5
Diabetes	1.02	0.55–1.90	12 / 36.9
No diabetes	Ref	-	60 / 224.5
Cancer	*3.15*	1.44–6.91	7 / 9.9
No cancer	Ref	-	65 / 251.5
Cardiovascular disease	1.02	0.66–1.56	33 / 108.0
No cardiovascular disease	Ref	-	39 / 153.4
History of SARS-CoV-2 infection	*0.10*	0.04–0.27	6 / 117.6
No history of SARS-CoV-2 infection	Ref	-	66 / 143.9
VOC Omicron BA.2	3.01	0.99–9.13	31 / 111.7
VOC Omicron BA.5	*17.95*	2.35-137.13	12 / 70.3
VOC Omicron BA.1	Ref	-	29 / 79.4

Hazard ratios with 95% confidence intervals were estimated using multivariable Cox proportional hazards models for breakthrough infection, adjusted for covariates and accounting for clustering at the nursing home level. Event counts and person-time are reported for descriptive purposes only; hazard ratios are not directly calculated from these incidence rates. Hazard ratios shown in italics are statistically significant (*p* < 0.05)

^a^Reference for nursing home residents: female, no hypertension, no respiratory disease, no diabetes, no cancer, no cardiovascular disease, no history of SARS-CoV-2 infection prior to primary course vaccination and a breakthrough infection during the Omicron BA.1 wave (31/12/2021–03/03/2022). Omicron BA.2 was the VOC during 04/03/2022–14/06/2022 and Omicron BA.5 during 15/06/2022–25/10/2022

A sensitivity analysis was performed in all breakthrough infections including symptomatic and asymptomatic and is shown in Supplementary Figs. 2 and 3 and Supplementary Tables 3, 4, and 5. The same predictors remained significant. In NHR within SCOPE2, the Omicron BA.5 wave (HR 10.39, 95% CI 0.95–113.70) was no longer identified as a predictor of breakthrough infection, whereas the Omicron BA.2 wave, previously not identified as a predictor, was now significantly associated with breakthrough infection (HR 2.76, 95% CI 1.02–7.52).

### Pre-infection antibody concentrations in breakthrough cases versus controls

**Fig. 6:**
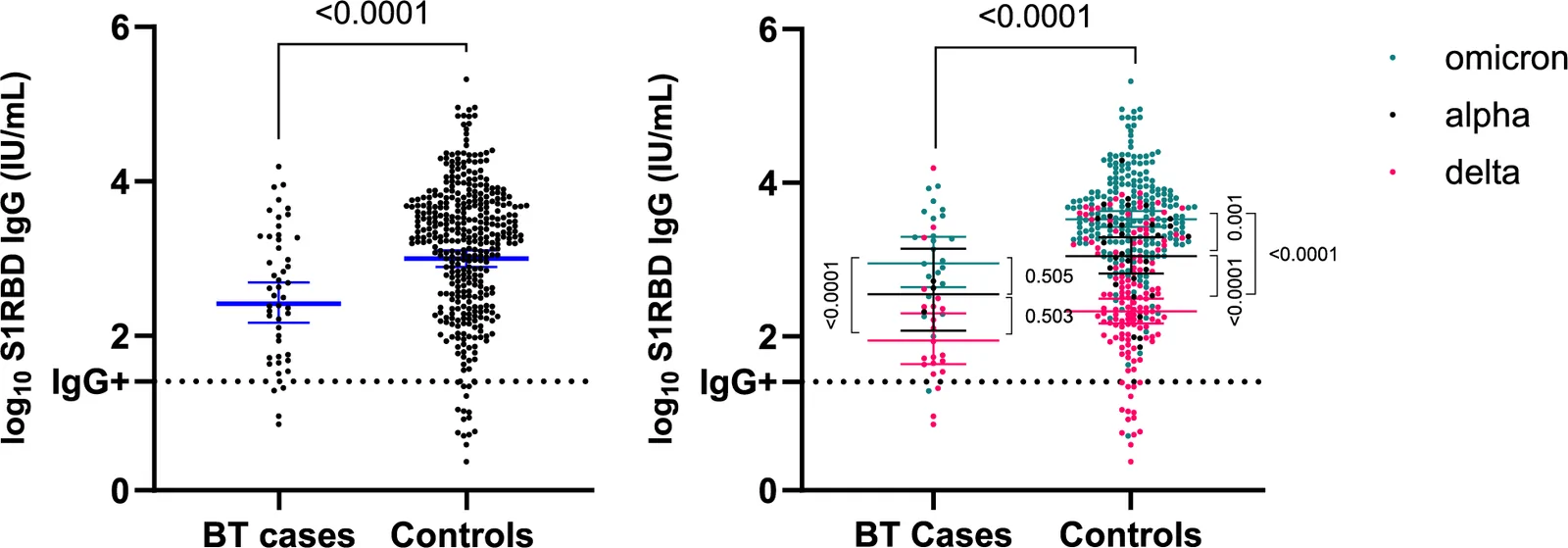
Left: Pre-infection SARS-CoV-2 anti-spike 1 receptor binding domain (S1-RBD) IgG antibody concentrations among breakthrough cases and matched controls. Right: Pre-infection S1-RBD IgG antibody concentrations among breakthrough cases and matched controls colored according to circulating variant, Belgium (2021–2022). Pre-infection S1-RBD IgG levels were defined as the antibody level measured ≤ 30 days before a reported breakthrough infection. Black, red and green dots represent samples collected during the Alpha, Delta and Omicron wave, respectively. Lines with error bars represent the geometric mean with 95% confidence intervals (per variant for the figure on the right). Dashed horizontal lines represent the cut-off for S1-RBD IgG positivity (26 IU/mL). BT: breakthrough

Geometric mean pre-infection S1-RBD IgG concentrations were statistically significantly lower among breakthrough cases (370 IU/mL; 95% CI 211–649) compared to matched controls (1434 IU/mL; 95% CI 1172–1754) (Fig. 6). The difference remained statistically significant when stratified by the Omicron and Delta wave (Supplementary Fig. 4). For both breakthrough cases and their controls, (pre-infection) geometric mean S1-RBD IgG concentrations were significantly higher during the Omicron wave than during the Delta wave (*p* < 0.0001). Within breakthrough cases, geometric mean pre-infection concentrations were comparable between NHR and NHS, suggesting similar thresholds for both groups (Supplementary Fig. 5).

### Peak vaccine response S1-RBD antibody concentrations in breakthrough cases versus controls

**Fig. 7:**
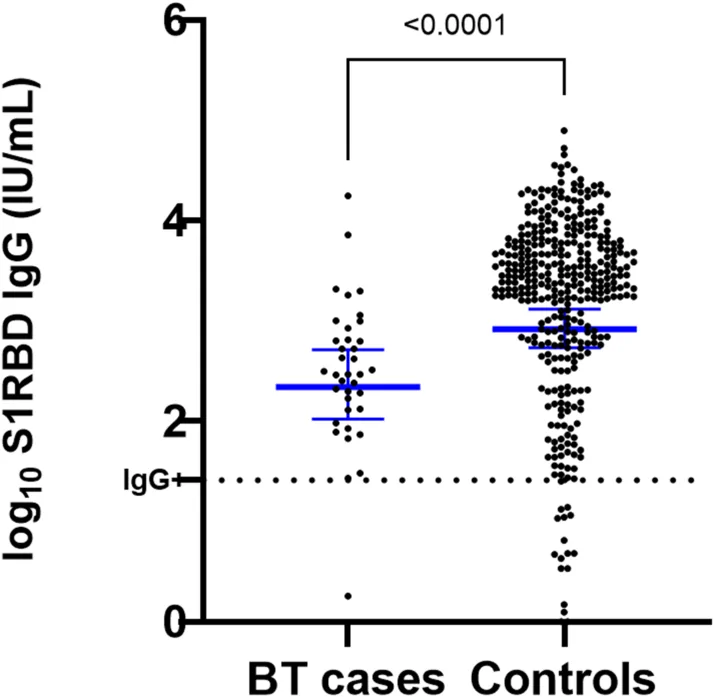
Peak vaccine response SARS-CoV-2 anti-spike 1 receptor binding domain (S1-RBD) IgG antibody concentrations (IU/mL) for breakthrough cases and matched controls. Peak vaccine response S1-RBD IgG concentrations were defined as the antibody concentrations measured during the first visit after the primary vaccination course (April 2021). Lines with error bars represent the geometric mean with 95% confidence intervals. Dashed horizontal lines represent the cut-off for S1-RBD IgG positivity (26 IU/mL). BT: breakthrough

Figure 7 shows peak vaccine response S1-RBD IgG concentrations among breakthrough cases and matched controls. The geometric mean peak vaccine response S1-RBD IgG concentration was significantly lower among breakthrough cases (313 IU/mL, 95% CI 182–536) than among matched controls (1588 IU/mL, 95% CI 1284–1966) (*p* < 0.0001). Moreover, the geometric mean peak vaccine response S1-RBD IgG concentration among breakthrough cases were significantly lower among NHR compared to NHS (*p* = 0.0158) (Supplementary Fig. 6).

### Pre-infection and peak vaccine response antibody thresholds

**Fig. 8:**
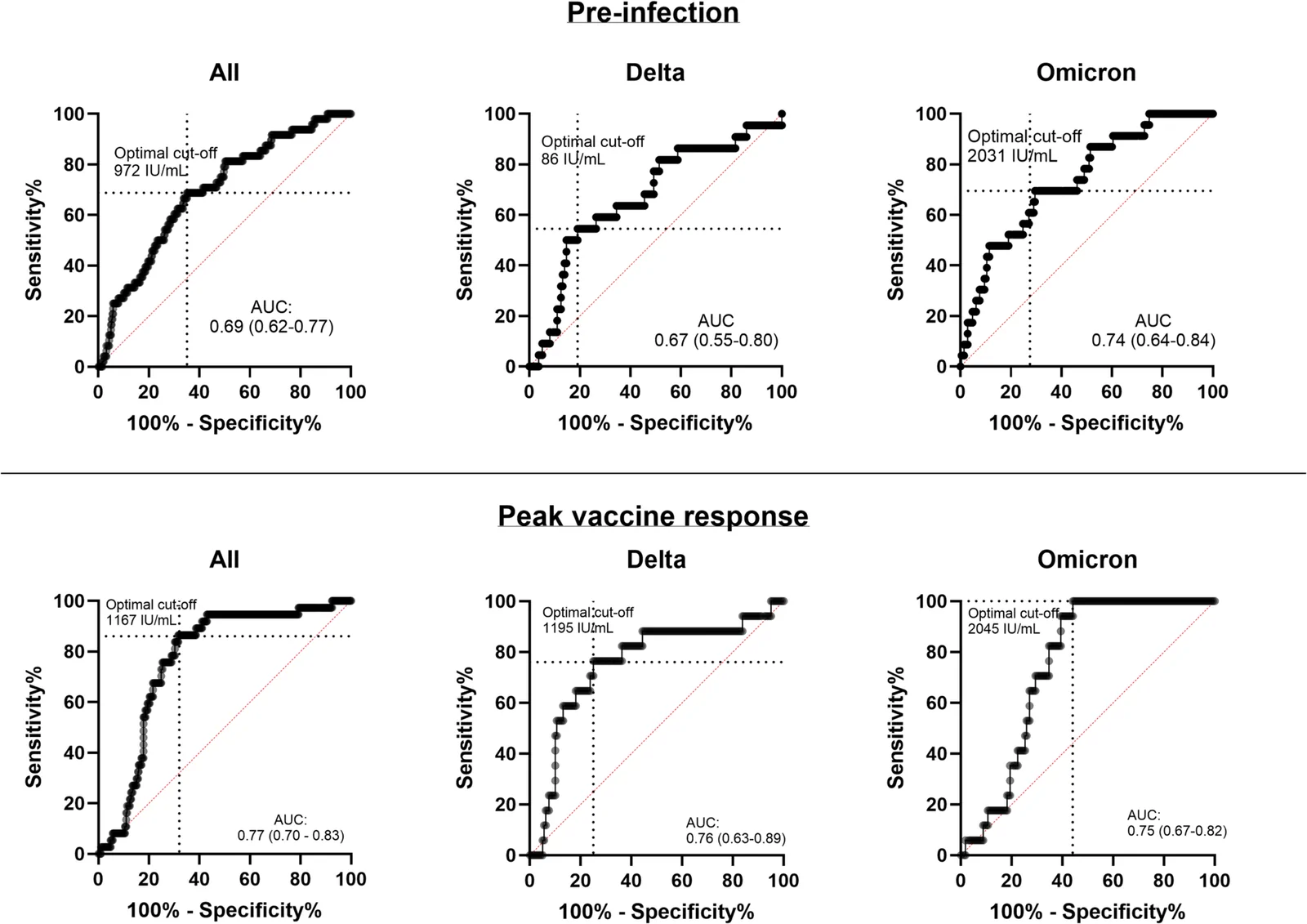
Receiver Operating Characteristic (ROC) curves for pre-infection data (above horizontal line) and peak vaccine response data (below horizontal line), for all samples and separate for samples collected during the Delta and Omicron wave

Figure 8 presents ROC curves evaluating how well S1-RBD IgG antibody concentrations distinguish individuals who developed a breakthrough infection from those who did not, overall and during the Delta and Omicron wave, for both pre-infection and peak vaccine response antibody concentrations.

For pre-infection antibody concentrations, the AUC values ranged from 0.67 to 0.74 across the three models, indicating acceptable to good classification performance. The optimal (based on Youden-index) pre-infection antibody thresholds for classification between breakthrough cases and their controls were 972 IU/mL for all breakthrough infections combined, 86 IU/mL for breakthrough infections during the Delta wave and 2031 IU/mL for breakthrough infections during the Omicron wave.

For peak vaccine response antibody concentrations, the AUC values ranged from 0.75 to 0.76 across the three models, again indicating good classification. The optimal peak vaccine response thresholds for classification between subjects who were going to experience a breakthrough infection and those who did not were 1167 IU/mL for all breakthrough infections combined, 1195 IU/mL for breakthrough infections during the Delta wave and 2045 IU/mL for breakthrough infections during the Omicron wave. The corresponding sensitivities and specificities for each threshold are shown in Table 4.

**Table 4:** Optimal pre-infection and peak vaccine response antibody for classification between breakthrough cases and controls

		Optimal S1-RBD IgG threshold (IU/mL)	Sensitivity^a^ (95% CI)	Specificity^b^ (95% CI)
Pre-infection	All	972	69 (55–80)	65 (60–70)
	Delta	86	55 (35–73)	81 (73–87)
	Omicron	2031	70 (49–84)	70 (64–76)
Peak vaccine response	All	1167	86 (72–94)	68 (63–72)
	Delta	1195	76 (53–90)	75 (68–81)
	Omicron	2045	100 (82–100)	56 (48–63)

^a^ Sensitivity refers to the ability to detect breakthrough disease in individuals with an S1-RBD IgG level below the optimal threshold. ^b^ Specificity of the assay refers to the ability to identify individuals with an S1-RBD IgG level above the optimal threshold without breakthrough disease

### Net benefit analysis of the antibody thresholds for protection

To evaluate the clinical utility of the established thresholds in vaccine intervention decision-making, a net benefit analysis was performed on the antibody thresholds established by the ROC analysis. For each antibody threshold, the predicted infection probability, net benefit, FP/TP ratio and the risk threshold range for which the net benefit was higher than vaccinating all or none is presented in Table 5. Decision curves depicting the net benefit of using antibody thresholds across different high-risk thresholds are shown in Supplementary Fig. 7. Predicted infection probabilities ranged from 6% to 17% across antibody thresholds, indicating the chance of infection at the respective threshold. FP/TP ratios ranged from moderate to high, with the highest observed for the pre-infection antibody thresholds for infections during the delta wave, meaning that for every subject correctly classified as unprotected, 8.83 subjects are incorrectly classified as unprotected. For all antibody thresholds, the net benefit of using the antibody threshold to decide for vaccine intervention was higher than treating all or none at risk thresholds ranging from 0.11% to 54.71% (Table 5), reflecting the added benefit of using antibody thresholds as a clinical decision tool. In all evaluated scenarios (pre- and peak vaccine response across variants), the antibody model yielded the highest net benefit at low threshold probabilities ranging between 0.94% and 13.26%, suggesting that antibody thresholds are most clinically useful when the risk level at which intervention is desired is low, such as in the case of prevention through vaccination.

**Table 5:** Infection probability, net benefit, false positive/true positive (FP/TP) ratio, risk threshold range for antibody thresholds

	All					Delta					Omicron				
	Antibody threshold (IU/mL)	Predicted infection probability (%)	Net benefit	FP/TP	Risk threshold range^a^(%)	Antibody threshold (IU/mL)	Predicted infection probability (%)	Net benefit	FP/TP	Risk threshold range^a^ (%)	Antibody threshold (IU/mL)	Predicted infection probability (%)	Net benefit	FP/TP	Risk threshold range^a^ (%)
Pre-infection	972	11	0.005	5.71	0.82–26.08	86	17	0.000	8.83	0.94–13.26	2031	10	0.010	3.81	0.11–54.71
Peak vaccine response	1167	8	0.010	6.67	0.44–28.57	1167	9	0.010	5.66	0.47–47.72	2045	6	0.015	7.99	0.47–23.13

^a^ Risk threshold range at which the net benefit of using antibody thresholds in clinical decision making is higher than vaccinating all or none

## Discussion

### Summary of key findings

In this study, we investigated the incidence, risk factors, and antibody thresholds of breakthrough infections among Belgian NHR and NHS in 2021 and 2022 (latter NHR only). In 2021, incidences were 2.4 (95% CI 1.59–3.46) per 100 person years in NHR and 10.7 (95% CI 8.6–13.2) in NHS. The incidence in NHR increased to 10.6 (95% CI 8.3–13.3) per 100 person years in 2022 during the circulation of highly transmissible Omicron subvariants. Prior SARS-CoV-2 infection was protective in both groups, while younger age and infection during the Delta wave increased breakthrough risk in NHS, and the Omicron BA.5 wave was linked to higher risk in NHR in 2022. Breakthrough cases had significantly lower pre-infection and peak vaccine response S1-RBD IgG concentrations than matched controls, suggesting a protective role of these antibodies. Both pre-infection and peak vaccine response antibody thresholds were identified, with higher protective thresholds for Omicron-related infections than Delta, highlighting the variant-specific nature of these thresholds. Net benefit analysis revealed a modest benefit of using antibody thresholds in guiding vaccine intervention compared to vaccinating all or none, mainly at low risk thresholds.

### Comparison with literature

Breakthrough infections were observed in a minority of fully vaccinated NHR and NHS and occurred less frequently than in the pre-vaccination period, when seroprevalence among unvaccinated NHR and NHS was 21% (95% CI 18–23) in spring 2020 [26], and 17.1% (95% CI 14.9–19.5) in fall 2020 [27].

Our findings align with infection rates reported in NHR during a similar time period [28] and with rates observed among NHS in other healthcare settings, such as the 9.7% breakthrough infection rate reported among hospital healthcare workers between January 2021 and February 2022 [29]. Furthermore, the frequency of breakthrough infections aligns with findings from a nationwide Belgian study of over eight million fully vaccinated adults, which reported a 4.6% breakthrough infection rate between February and December 2021 [5]. This study included both symptomatic and asymptomatic cases, whereas our study focused on symptomatic infections. Although we did not assess disease severity, previous studies reported hospitalization and/or mortality rate of approximately 8% among NHR with breakthrough infections [28, 30], considerably lower than during the pre-vaccination period (hospitalization 10–58%, mortality 23–31%) [31]. Additionally, Belgian surveillance data showed sharp declines in both hospital admissions and mortality following vaccination [32]. Importantly, this indicates reduced disease severity despite ongoing infections among NHR.

Our previous work showed that NHR develop lower antibody levels after COVID-19 vaccination than younger individuals, such as NHS [15, 16, 24], which would suggest a higher risk of breakthrough infection. However, we observed the opposite, with higher breakthrough infection rates among NHS, consistent with earlier findings of more reinfections in unvaccinated NHS [26]. Additionally, pre-infection antibody levels among breakthrough cases were similar between NHR and NHS, suggesting a comparable protective threshold and indicating that exposure risk may outweigh vaccine-induced immunity. Younger age increased the risk of breakthrough infection in NHS, likely reflecting greater social exposure, consistent with findings in the general Belgian population and other studies [5, 33, 34]. Differences in booster timing relative to the onset of the fourth wave (October 2021) may have contributed to the differences in breakthrough infection rates between NHR and NHS, as NHR were prioritised earlier in the booster campaign, whereas NHS became eligible later (from 27 November 2021) [5, 35].

In our study, prior infection was protective against breakthrough infection in both NHS and NHR, consistent with previous studies [36, 37], supporting the concept of hybrid immunity [38–42]. Our earlier work showed that prior infection was associated with higher and more durable post-vaccination antibody levels in in both groups [4, 15, 24], suggesting a mediating role for antibodies. Consistently, breakthrough cases had lower pre-infection S1-RBD IgG concentrations than controls, reinforcing evidence that S1-RBD IgG is a key immune correlate of protection against breakthrough infection [7, 10, 12, 13, 43, 44]. Neutralizing antibodies have been identified as key immune correlates for protection against breakthrough infections [6, 7, 11], while binding antibodies are strongly correlated with neutralizing antibodies, and increasingly suggested as surrogate markers [45–47]. Although some studies report associations between binding antibody concentrations and breakthrough infections [10, 12, 13], others do not [14, 48, 49], likely reflecting differences in study design, circulating variants, vaccine types, or sampling timing. Despite these inconsistencies, the ease of measuring binding antibodies makes them attractive for monitoring immune protection. Notably, some individuals with high antibody levels still experienced breakthrough infection in our study, suggesting that additional immune mechanisms, such as T-cell responses, might contribute to protection against infection [11, 50]. However, these were not assessed here.

Certain SARS-CoV-2 variants exhibit immune escape, allowing breakthrough infections despite vaccination. Consistent with our findings in NHR the Belgian population data reported incidence rates were higher during the Delta than the Alpha wave [5]. Pre-infection antibody concentrations differed by variant, with higher levels in breakthrough cases during Omicron than Delta, and intermediate levels during Alpha. ROC analyses confirmed variant-specific protective antibody thresholds, with higher threshold required during the Omicron than during the Delta wave, consistent with Omicron’s higher level of immune evasion and reduced neutralizing activity [51, 52]. These variant-dependent thresholds complicate the definition of a universal correlate of protection and may require adaptation for future variants. Higher antibody levels among controls during Omicron compared with Delta likely reflect differences in timing, as Omicron emerged shortly after booster vaccination, while Delta occurred during antibody waning. In contrast, Smoot et al. found that lower antibody levels were associated with subsequent breakthrough infection among NHR during the Delta wave, but not during Omicron. This discrepancy may be explained by their cross-sectional study design [53].

Several studies have attempted to define protective antibody thresholds against SARS-CoV-2 infection, most often expressed in binding antibody units (BAU)/mL, with reported values ranging widely from 60 to 6967 BAU/mL [12, 52, 54, 55]. As 1 IU/mL equals 1 BAU/mL, our results are directly comparable, as all measure binding antibodies [21, 56]. In a longitudinal study, McGee et al. found that 82% of breakthrough infections occurred at anti-spike titers below 3000 arbitrary units (AU)/mL [12]. The broad variation in thresholds likely reflects differences in quantification methods, antibody targets, populations, outcome definitions, timing, and circulating variants, highlighting the challenge of establishing a universal protective threshold.

Our net benefit analysis indicated that applying antibody thresholds to guide vaccine decisions offers added value at low risk thresholds, particularly when the acceptable intervention risk ranges from approximately 1% to 13%. This approach may be most relevant in low-risk settings or when resources or vaccine supply are limited. Nevertheless, the observed net benefits were modest, and decision-making may be better informed by outcomes such as severe disease or hospitalization, rather than symptomatic breakthrough infection. Therefore, the proposed antibody thresholds should be considered context-dependent and indicative rather than definitive.

### Strengths and limitations

This prospective study assessed breakthrough infections after COVID-19 vaccine in a nationwide population of NHR and NHS. Key strengths are the inclusion of both NHR and NHS, the large sample size and randomized selection. Several limitations should be noted. Data were collected using questionnaires, and breakthrough infection were (self-)reported, potentially leading to underreporting due to recall bias. Although hypertension and cardiovascular disease were recorded as separate comorbidities, misclassification may have occurred. Changes in preventive measures, testing policies, and lack of detailed exposure data may also have influenced infection rates.

Time since vaccination was not adjusted for, as primary vaccinations and booster doses were largely administered within the same, centrally organised time windows, resulting in limited variability. Furthermore, the VOC was inferred based on the dominant circulating strains rather than molecular confirmation, creating uncertainty during periods of variant co-circulation. In SCOPE2, the smaller NHR sample size limited the precision of some estimates, as reflected by wide CI for certain VOC-specific effects. Additionally, the study may have been underpowered to detect associations with individual comorbidities (e.g., cancer, diabetes), due to the limited number of events in these subgroups. As a result, the absence of statistically significant associations for specific comorbidities should be interpreted with caution.

Due to the limited number of cases and DBS samples collected shortly before infection, adjustment for covariates through multivariable analysis or separate antibody analyses for NHS and NHR were not feasible. Moreover, funding constraints prevented antibody measurements at all timepoints, precluding inclusion of S1-RBD IgG as predictors in the Cox models Although DBS-based S1-RBD IgG measurements were previously validated, they may slightly overestimate serum concentrations [22]. Finally, NHS reported symptoms more frequently than NHR, which may reflect reporting bias or lower symptom recognition in older adults [57]. Sensitivity analyses including symptomatic and asymptomatic infections yielded largely similar predictors. However, among NHR in SCOPE2, infection during the Omicron BA.5 wave was no longer significant, whereas the Omicron BA.2 wave emerged as significant, likely reflecting a higher number of events in a small sample.

### Practical implications

Our findings underscore the importance of monitoring breakthrough infections in high-risk groups such as NHR and NHS, particularly in the context of emerging variants. Increased symptomatic breakthrough infections among NHR during the Omicron BA.5 wave underscores the need for targeted preventive strategies, while higher rates among NHS in 2021 suggest that younger, more socially active populations may also require tailored interventions. As vaccine effectiveness may wane over time and vary by variant, continued surveillance is essential to identifying individuals at higher risk and to inform targeted booster strategies.

Our study gained more insight into two key antibody thresholds: pre-infection levels, which reflect short-term infection risk and may support timely preventive measures, and peak post-vaccination responses, which may predict future breakthrough infections. Together these findings contribute to efforts to refine antibody-based risk stratification, although further research is needed to establish variant-specific correlates of protection and adapt thresholds to evolving infection dynamics.

## Conclusion

Breakthrough infections were uncommon among fully vaccinated NHR and NHS, with younger age increasing risk among NHS. Prior natural SARS-CoV-2 infection was found to be protective in both groups, strengthening the concept of hybrid immunity. Additionally, breakthrough cases had significantly lower pre-infection and peak vaccine response S1-RBD IgG antibody concentrations compared to matched controls, highlighting the role of (binding) antibodies in mediating protection. The risk of breakthrough infection and antibody thresholds varied by variant. Our findings contribute to the understanding of COVID-19 vaccine-induced protection in NHR and NHS, and its association with antibody levels, enabling the optimization of vaccination strategies for these vulnerable populations.

## Supplementary Information

Supplementary Material 1.

## Data Availability

The sponsor of this study (Sciensano) shares ownership of the data. Therefore, the data are available upon request and after permission of the sponsor.

## Abbreviations

AUC: Area Under the Curve
COVID-19: COronaVIrus Disease 2019
CI: Confidence Interval
DBS: Dried Blood Spots
FP: False Positive
IgG: Immunoglobin G
IU/mL: International Units/mL
IQR: InterQuartile Range
N: Number
NH: Nursing Home
NHR: Nursing Home Residents
NHS: Nursing Home Staff
OD: Optical Density
PCR: Polymerase Chain Reaction
ROC: Receiver Operating Characteristic
SARS-CoV-2: Severe Acute Respiratory Syndrome COronaVirus 2
S1-RBD: Spike 1-Receptor Binding Domain
TP: True Positive
VOC: Variant of Concern
WHO: World Health Organization
