# Supplementary material for: SARS-CoV-2 breakthrough infection incidence, risk factors, and antibody correlates in Belgian nursing homes residents and staff (2021–2022)
Source: Arch Public Health. 2026 Jun 24;84:198. doi: 10.1186/s13690-026-01939-7 (PMC13555862; doi:10.1186/s13690-026-01939-7)
Supplement: Supplementary file 1 [file 13690_2026_1939_MOESM1_ESM.docx]

**Supplementary File**

**Explanation matched control dataset**

In the SCOPE2 study, DBS were immediately analyzed for S1RBD IgG after each study visit. Therefore, a database with S1RBD IgG concentrations for every collected sample already existed. In the SCOPE1 study, DBS were collected in addition to SARS-CoV-2 antibody rapid tests. Here, DBS were not immediately analyzed upon collection but stored at -20°C. However, subsets of interest were previously analyzed for previously published work [1]. In this way, a dataset with S1RBD IgG concentrations for a large number of DBS collected within SCOPE1 was already available too. In the current study, matched controls were searched within a combined version of the available datasets from SCOPE1 and SCOPE2. This dataset included the S1RBD IgG concentrations for n=3130 DBS collected at different visits (Supplementary Table 1).

**Supplementary Table 1. Overview of the number of samples included in the dataset wherein matched controls were searched.**

| **Study** | **Visit** | **Month** | **n** |
| --- | --- | --- | --- |
| SCOPE1 | 2 | April 2021 | 796 |
| SCOPE1 | 3 | June 2021 | 696 |
| SCOPE1 | 4 | August 2021 | 665 |
| SCOPE1 | 5 | October 2021 | 182 |
| SCOPE1 | 6 | December 2021 | 430 |
| SCOPE2 | 7 | March 2022 | 361 |

**Supplementary Table 2. Reported symptoms in breakthrough infections in nursing home residents and staff, February 2021- September 2022, Belgium.**

| **Symptoms, n(%)** | **Nursing Home Residents (n=100)** | Missing | **Nursing Home Staff (n=87)** | Missing |
| --- | --- | --- | --- | --- |
| **Cough** | 57 (57%) | 30 | 60 (69%) | 20 |
| **Dyspnoea** | 13 (13%) | 65 | 38 (44%) | 41 |
| **Chest pain** | 3 (3%) | 73 | 22 (25%) | 53 |
| **Loss of taste** | 1 (1%) | 74 | 45 (52%) | 28 |
| **Loss of smell** | 1 (1%) | 74 | 52 (60%) | 25 |
| **Worsening of chronic respiratory conditions** (e.g., COPD, asthma, chronic cough, etc.) | 2 (2%) | 74 | 7 (8%) | 63 |
| **Fever** | 30 (30%) | 57 | 33 (38%) | 43 |
| **Myalgia** | 10 (10%) | 70 | 50 (57%) | 29 |
| **Fatigue** | 34 (34%) | 47 | 71 (82%) | 11 |
| **Runny nose** | 22 (22%) | 55 | 61 (70%) | 23 |
| **Throat ache** | 17 (17%) | 61 | 44 (51%) | 38 |
| **Headache** | 13 (13%) | 63 | 65 (75%) | 17 |
| **Loss of appetite** | 14 (14%) | 62 | 26 (30%) | 46 |
| **Diarrhoea** | 12 (12%) | 67 | 16 (18%) | 56 |
| **Confusion** | 4 (4%) | 71 | 3 (3%) | 67 |
| **Collapse** | 2 (2%) | 73 | 0 | 69 |

*Symptoms reported in symptomatic breakthrough infections in nursing home residents and staff.*

**Supplementary Figure 1***.* Cumulative incidence of breakthrough infections among fully vaccinated nursing home residents in SCOPE2, 2022, Belgium. Shaded areas indicate 95% confidence intervals. Full vaccination was defined as 14 days post-primary course.

**Sensitivity analyses of all breakthrough infections (with and without symptoms)**

Sensitivity analysis was performed in all breakthrough infections (with and without symptoms). To ensure that these infections were not due to persistent positivity from a prior infection, we verified that none of the cases had tested positive in the 90 days preceding their breakthrough infection. In SCOPE1, a total of 159 (6%) breakthrough infections were observed, 55 (3%) among NHR and 104 (9%) among NHS. The majority of infections among staff still occurred in November 2021 (Supplementary Figure 2). In SCOPE2, 104 (22%) breakthrough infections were reported among NHR. Most infections occurred in January 2022, with a substantial number still reported in March 2022 (Supplementary Figure 3). The same predictors remained significant as in the main analysis assessing symptomatic breakthrough infections (Supplementary Tables 3, 4, 5). In NHR within SCOPE2, cancer and the Omicron BA.5 variant were also identified as predictors of breakthrough infection, likely due to the higher number of events.

**Supplementary Figure 2**. Frequency of all breakthrough infection (with and without reported symptoms) in fully vaccinated nursing home residents and staff between February and December 2021, Belgium.

**Supplementary Figure 3.** Frequency of all breakthrough infection (with and without reported symptoms) in fully vaccinated nursing home residents between January and August 2022, Belgium.

**Supplementary Table 3. Sensitivity analysis (including symptomatic and asymptomatic infections):** predictors associated with a breakthrough infection in nursing home staff^a^ February – December 2021, Belgium (Cox proportional hazard model).

| **Predictors** | **Hazard Ratio** | **95% Confidence Interval** | **Events/person-years** |
| --- | --- | --- | --- |
| Age | 0.98 | 0.96 - 0.99 | - |
| Male | 0.75 | 0.41 - 1.40 | 12 / 117.0 |
| Female | Ref | - | 87 / 646.6 |
| No comorbidities | 0.93 | 0.50 - 1.71 | 82 / 621.7 |
| ≥1 comorbidity | Ref | - | 17 / 142.0 |
| History of SARS-CoV-2 infection | 0.31 | 0.18 - 0.54 | 13 / 248.7 |
| No history of SARS-CoV-2 infection | Ref | - | 86 / 514.9 |
| Delta wave | 4.77 | 1.61 - 14.16 | 87 / 423.7 |
| Alpha wave | Ref | - | 12 / 339.9 |

*Hazard ratios with 95% confidence intervals were estimated using multivariable Cox proportional hazards models for breakthrough infection, adjusted for covariates and accounting for clustering at the nursing home level. Event counts and person-time are reported for descriptive purposes only; hazard ratios are not directly calculated from these incidence rates. Underlined hazard ratios are statistically significant (p<0.05).*

*^a^Reference for nursing home staff: female, presence of a comorbidity, no history of SARS-CoV-2 infection prior to primary course vaccination, and a breakthrough infection during the Alpha wave (15/02/2021–27/06/2021). Delta was the VOC during 28/06/2021–30/12/2021.*

**Supplementary Table 4. Sensitivity analysis (including symptomatic and asymptomatic infections):** predictors associated with a breakthrough infection in nursing home residents^a^ February – December 2021, Belgium (Cox proportional hazard model).

| **Predictors** | **Hazard Ratio** | **95% Confidence Interval** | **Events/person-years** |
| --- | --- | --- | --- |
| Age | 0.97 | 0.93 - 1.01 | - |
| Male | 0.71 | 0.34 - 1.48 | 12 / 277.3 |
| Female | Ref | - | 41 / 863.6 |
| Hypertension | 0.87 | 0.56 - 1.36 | 16 / 390.1 |
| No hypertension | Ref | - | 37 / 750.8 |
| Respiratory disease | 1.17 | 0.60 - 2.29 | 7 / 124.3 |
| No respiratory disease | Ref | - | 46 / 1016.6 |
| Immune disorder | 1.29 | 0.23 – 7.15 | 2 / 27.1 |
| No immune disorder | Ref | - | 51 / 1113.7 |
| Diabetes | 0.84 | 0.37 - 1.90 | 8 / 202.2 |
| No diabetes | Ref | - | 45 / 938.7 |
| Cancer | 1.51 | 0.32 – 7.22 | 3 / 49.2 |
| No cancer | Ref | - | 50 / 1091.6 |
| Cardiovascular disease | 1.16 | 0.73 - 1.83 | 22 / 465.8 |
| No cardiovascular disease | Ref | - | 31 / 675.1 |
| History of SARS-CoV-2 infection | 0.09 | 0.02 - 0.36 | 3 / 443.6 |
| No history of SARS-CoV-2 infection | Ref | - | 50 / 697.2 |

*Hazard ratios with 95% confidence intervals were estimated using multivariable Cox proportional hazards models for breakthrough infection, adjusted for covariates and accounting for clustering at the nursing home level. Event counts and person-time are reported for descriptive purposes only; hazard ratios are not directly calculated from these incidence rates. Underlined hazard ratios are statistically significant (p<0.05).*

*^a^Reference for nursing home residents: female, no hypertension, no respiratory disease, no immune disorder, no diabetes, no cancer, no cardiovascular disease, and no history of SARS-CoV-2 infection prior to primary course vaccination.*

**Supplementary Table 5. Sensitivity analysis (including symptomatic and asymptomatic infections):** predictors associated with a breakthrough infection in nursing home residents^a^ January – September 2022, Belgium (Cox proportional hazard model).

| **Predictors** | **Hazard Ratio** | **95% Confidence Interval** | **Events/person-years** |
| --- | --- | --- | --- |
| Age | 1 | 0.98 - 1.01 | - |
| Male | 0.94 | 0.68 - 1.30 | 30 / 63.5 |
| Female | Ref | - | 74 / 192.8 |
| Hypertension | 1.47 | 0.92 - 2.36 | 47 / 87.9 |
| No hypertension | Ref | - | 57 / 168.4 |
| Respiratory disease | 1.12 | 0.56 - 2.25 | 12 / 25.4 |
| No respiratory disease | Ref | - | 92 / 230.8 |
| Diabetes | 1.08 | 0.68 - 1.72 | 18 / 36.0 |
| No diabetes | Ref | - | 86 / 220.3 |
| Cancer | 3.17 | 1.78 – 5.63 | 10 / 9.4 |
| No cancer | Ref | - | 94 / 246.9 |
| Cardiovascular disease | 0.93 | 0.64 - 1.33 | 47 / 105.8 |
| No cardiovascular disease | Ref | - | 57 / 150.4 |
| History of SARS-CoV-2 infection | 0.08 | 0.04 - 0.18 | 7 / 117.5 |
| No history of SARS-CoV-2 infection | Ref | - | 97 / 138.8 |
| Omicron BA.2 | 2.76 | 1.02 – 7.52 | 42 / 109.1 |
| Omicron BA.5 | 10.39 | 0.95 – 113.70 | 14 / 70.1 |
| Omicron BA.1 | Ref | - | 48 / 77.1 |

*Hazard ratios with 95% confidence intervals were estimated using multivariable Cox proportional hazards models for breakthrough infection, adjusted for covariates and accounting for clustering at the nursing home level. Event counts and person-time are reported for descriptive purposes only; hazard ratios are not directly calculated from these incidence rates. Underlined hazard ratios are statistically significant (p<0.05).*

*^a^Reference for nursing home residents: female, no hypertension, no respiratory disease, no diabetes, no cancer, no cardiovascular disease, no history of SARS-CoV-2 infection prior to primary course vaccination and a breakthrough infection during the Omicron BA.1 wave (31/12/2021–03/03/2022). Omicron BA.2 was the VOC during 04/03/2022–14/06/2022 and Omicron BA.5 during 15/06/2022–25/10/2022.*

**Supplementary Figure 4**. Pre-infection S1RBD IgG concentrations among Delta (left) and Omicron (right) breakthrough cases and matched controls. Lines with error bars represent the geometric mean with 95% confidence intervals. BT: breakthrough.

**Supplementary Figure 5**. Pre-infection S1RBD IgG concentrations among breakthrough cases: nursing home residents (NHR) versus nursing home staff (NHS). Lines with error bars represent the geometric mean with 95% confidence intervals.

**Supplementary Figure 6**. Peak vaccination S1RBD IgG concentrations among breakthrough cases: nursing home residents (NHR) versus nursing home staff (NHS). Lines with error bars represent the geometric mean with 95% confidence intervals.

**Supplementary Figure 7. Decision curves for pre-infection and peak vaccine response data for all variants and separate for breakthrough infections during the Delta and Omicron wave.**

***References:***

1.	Meyers, E.; De Rop, L.; Engels, F.; Gioveni, C.; Coen, A.; De Burghgraeve, T.; Digregorio, M.; Van Ngoc, P.; De Clercq, N.; Buret, L.; et al. Follow-Up of SARS-CoV-2 Antibody Levels in Belgian Nursing Home Residents and Staff Two, Four and Six Months after Primary Course BNT162b2 Vaccination. Vaccines [Internet]. 2024; 12(8).
